# Comparative Study of Abiotic Stress Factors on GC-MS-Detected Phytoconstituents of *Aloe greatheadii* var: davyana Using Heat Map and Hierarchical Clustering Dendrogram

**DOI:** 10.1155/2022/5365024

**Published:** 2022-01-05

**Authors:** Denga Nthai, Vuyisile Samuel Thibane, Sechene Stanley Gololo

**Affiliations:** Department of Biochemistry, Sefako Makgatho Health Sciences University, P.O. Box 235, Medunsa, 0204 GA-Rankuwa, South Africa

## Abstract

*Aloe greatheadii* var. davyana or spotted aloe is indigenous to South Africa and widely distributed in the northern provinces. The plant has a vast ethnopharmacological application which is mostly attributed to its phytochemical content. The aim of the study was to examine the effect of abiotic stress factors on the plant's phytochemical content. The phytochemical content of *A. greatheadii* hexane extracts from four different provinces (Limpopo, Mpumalanga, Gauteng, and North West), harvested from the wild at varied altitudes, rainfall patterns, and soil types, was examined using gas chromatography-mass spectra (GC-MS). The phytochemical content of hexane extracts from the four South African provinces was analysed using heat map analysis and hierarchical clustering dendrogram. The phytochemical content of *A. greatheadii* hexane extracts was composed of fatty acids, alkanes, benzene, carboxylic acids, ketones, phytosterols, and vitamins. Eicosane, henicosane, and [(2S)-2-[(2R)-4-hexadecanoyloxy-3-hydroxy-5-oxo-2H-furan-2-yl]-2-hydroxyethyl] hexadecanoate were the only compounds detected in all samples from the four provinces. The concentration levels of 2-(((2-ethylhexyl)oxy)carbonyl) benzoic acid, beta-sitosterol, tritetracontane, and ethyl 13-methyltetradecanoate were closely related and expressed a low clustering distance amongst the samples. Variations in soil pH, soil type, and rainfall patterns were detected and differed in the four provinces. The different abiotic stress factors affected the biochemical pathways for the different compounds, with conditions in Gauteng being less favourable for many of the compounds detected. Abiotic stress factors have shown to influence phytochemical biochemical pathways and quantity. *Aloe greatheadii* plants can be selected based on location seemingly due to the variations that persist in their phytochemical content.

## 1. Introduction

*Aloe greatheadii* var. davyana (spotted aloe) belongs to the family Asphodelaceae. *Aloe greatheadii* is a winter flowering plant characterized by a stemless, solitary rosette of greyish-green succulent leaves with elongated whitish spots and sharp reddish-brown teeth along the margins. The plant is indigenous to South African and is widely distributed in the northern provinces [1]. The ethnobotanical use of the plant includes in the treatment of skin cancer, burns, eczema, psoriasis, high blood pressure, and diabetes. The reported wide traditional usage of the plant makes it an important plant sought after in natural product research. The phytochemical content of *A. greatheadii* is mainly composed of organic acids, alcohols, aldehydes, phenolic compounds, ketones, pyrimidines, alkaloids, phytosterols, and some fatty acids [2]. The health benefits of the plant can be associated with its phytochemical composition and antioxidant potential. *Aloe greatheadii* has been reported to exhibit antidiabetic potential by moderately increasing serum insulin and significantly decreasing the total cholesterol (TC): high-density lipoprotein-cholesterol (HDL-C) ratios in Wistar rats [3].

The wide distribution and biodiversity of plants exposes them to varying abiotic stress conditions such as varied altitudes, rainfall patterns, and soil types that are associated with those growing conditions [4]. Phytochemicals play a significant role in the plant defence system; however, variables in growing conditions would alter their concentration and composition [5]. The understanding of the role of abiotic stress plays an important factor in natural product research, due to the noticeable effects caused by climate change on the phytochemical composition of the world's vegetation [6]. Drought is one of the most encountered environmental stresses and can further be associated with water availability because of climate change. Plants growing at different altitudes, different rainfall patterns, and different soil types and pH experience variation in water availability and consequently altered biochemical pathways for phytochemicals [7]. The present study aimed at examining the effect of varied altitudes, rainfall patterns, and soil types on the phytochemical content of plant samples from Limpopo, Mpumalanga, Gauteng, and North West.

## 2. Materials and Methods

### 2.1. Plant Extract Preparation

Leaves of *Aloe greatheadii* were collected in the wild from four different locations using a convenient sampling method. Plants were identified by Dr. Bronwyn Egan, Department of Botany, University of Limpopo (UL), and a voucher specimen (UNIN 12990) was prepared and deposited in the Leach Herbarium (UL). Leaves were washed with sterile distilled water (H_2_O) to remove excess dirt. After washing, the leaves were firstly chopped into smaller pieces, dried at room temperature until constant dry weight, and finally, ground into a powder using a table-top blender. Five grams (5 g) of the ground plant materials were extracted with hexane using cold maceration with agitation for 24 h at room temperature. The solution was filtered using Whatman No.4 filter paper and allowed to dry under a stream of air. Extracts were stored in the fridge until analysis.

### 2.2. Abiotic Stress Factor (Altitude, Rainfall Pattern, and Soil pH) Consideration

The altitude and rainfall patterns of the collection sites were noted from online reports, SA Explorer (2000–2007) and Falling Rain Software, Ltd. (1996–2017). The pH of the soil was measured using a table-top pH meter.

### 2.3. Gas Chromatography Mass Spectra (GC-MS) Analysis

The GC-MS analysis of the hexane extracts of *A. greatheadii* was conducted using a SHIMADZU QP2010 SE GC-MS with an inert cap 5MS/SIL, silica capillary column (30 mm × 0.25 mm ID *X* 1 µmdf, composed of 100% dimethyl-poly-siloxane). An electron ionization system with an ionizing energy of 70 eV was used for detection. Helium gas (99.99%) was used as the carrier gas at a constant flow rate of 1 mm/min with an injection volume of 2 ul; injector temperature of 290°C; and ion-source temperature of 230°C. The oven temperature was set from 50°C (isothermal for 1 min), with an increase of 20°C/min to 180°C (isothermal for 5 min), and then increased to 240 C at 20°C/min, ending with an increase of 20ºC/min to 280°C (isothermal for 5 min). Mass spectra were taken at 70 eV; scan interval of 0.3 sec; and fragments from 50 to 700 m/z [8]. The software adopted to handle the mass spectra and chromatogram was GC-MS Solutions version 2.6. Identification of compounds was based on the mass spectra from the GC-MS and was performed using the compound database of the National Institute of Standard and Technology (NIST08) library. The mass spectra of unknown compounds were compared with those of components stored in the NIST08 library with the hit of 90% and above regarded as a positive match. The retention time, name, molecular weight, and molecular formula of the identified compounds were recorded.

### 2.4. Data Analysis

Data obtained were presented in the form of tables and histograms as mean ± standard deviation. Statistical analysis amongst the results of extracts of the plant samples from different provinces was conducted by one-way ANOVA using SPSS version 18 statistical package. Differences between groups were considered significant at *p* < 0.05. The experiments for data collection were run 3 times in duplicates.

## 3. Results and Discussion

### 3.1. Abiotic Stress Factor Consideration

**Figure 1:**
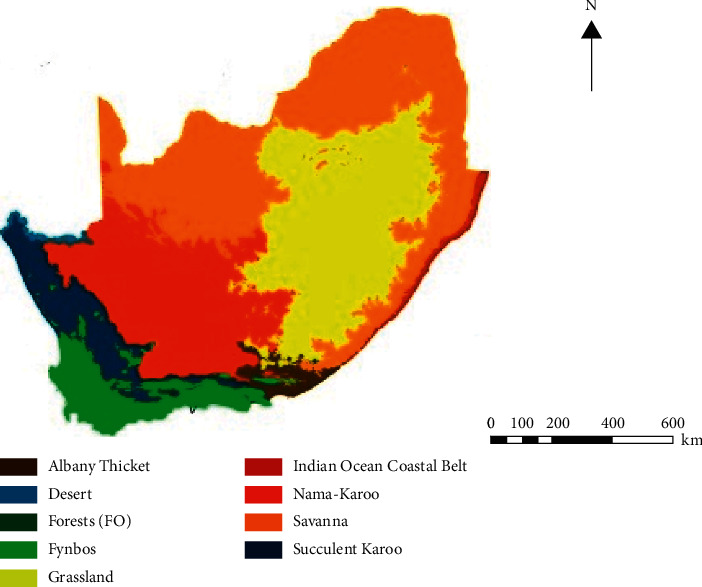
South African biome, characterized by the Albany thicket, desert, forests, fynbos, grassland, Indian Ocean coastal belt, Nama-Karoo, Savanna, and Succulent Karoo, adapted from [10, 11].

The results for the rainfall, soil pH, and soil type (abiotic stress factors) of the plant sample collection sites (Limpopo, Mpumalanga, Gauteng, and North-West) are presented in Table 1. The soil pH at the plant sample collection sites was found to be slightly acidic and varied between the four provinces at a range of 5.17–6.80. *Aloe* species are known to survive under such varying ecological conditions [9]. The Savannah and Grassland biome makes up a majority of the land in the four provinces (Figure 1). The sampling sites in Limpopo, Mpumalanga, and North-West shared both Savannah and grassland biome, while Gauteng was mainly characterized by grassland. The biomes in different geographic locations can have varying effects on the metabolite profiles of plants growing in the region [12, 13]. The soil type for the sampling sites was reported to be loam (Limpopo, Mpumalanga, and Gauteng) and clay (North-West). The reported annual rainfall average ranged from 389–540 mm with the North-West Province receiving the largest rainfall, while Limpopo received the least of the rain. The soil type and rainfall patterns play an important role in water retention and availability for the plants. The reported difference in abiotic stress factors of rainfall patterns, soil type, and soil pH will enable varying levels of secondary metabolites in *A. greatheadii.* This is significantly important, especially when looking at the biological activity of *A. greatheadii* and its commercial importance in natural products.

### 3.2. GC-MS Analysis

The n-hexane extracts of the leaves of *A. greatheadii* from four South African provinces were subjected to GC-MS analysis, and the detected compounds were identified by comparing their mass spectra with those stored in the NIST08 library. The detected compounds with 90% mass spectra similarity hits are shown in Table 2, and those below 90% similarity hit are not being reported on. Eicosane, heneicosane, and L-(+)-ascorbic acid 2, 6-dihexadecanoate were detected in samples from all provinces, whereas other compounds such as dodecanoic acid, tetradecanoic acid, methyl hexadecanoate, and ethyl hexadecanoate were detected in the Gauteng sample only. Furthermore, pentadecane, hexadecane, heptadecane, octadecane, ethyl 13-methyltetradecanoate, and tetratetracontane were detected in all the samples from the other provinces except for the Gauteng sample. Oxacycloheptadecane-2, 9-dione and eicosanoic acid were only detected in samples from Mpumalanga. 2-(((2-Ethylhexyl) oxy) carbonyl) benzoic acid, beta-sitosterol, and tritetracontane were detected in both the Mpumalanga and North-West samples. However, 1, 54-dibromotetrapentacontane was only present in the North-West sample. Dodecyl propan-2-yl sulfite and (Z)-icos-13-enoic acid were only detected in samples from Limpopo. The Gauteng Province has an altitude of 1246 m and with an estimated annual rainfall average of 519 mm. The soil type in Gauteng is loam with a recorded soil pH value of 5.98 where the sample was collected. The Gauteng Province sampling site is slightly elevated when compared to sites in Mpumalanga and North-West. The sampling site in Limpopo was much elevated than the site in Gauteng. However, the loam soil type was common for the sampling sites in Limpopo, Mpumalanga, and Gauteng, with the sampling site in North-West characterized by a clay soil type. The soil pH at the plant sample collection sites varied between the four provinces. It is interesting to note that the combination of different abiotic stress factors affected the biochemical pathways for the different compounds with conditions in Gauteng being less favourable for many of the compounds detected.

The results have demonstrated some variation in the GC-MS detectable compounds present in the leaves of *A. greatheadii* from the four South African provinces. The detection and identification of phytochemical compounds using GC-MS is a qualitative form of analysis with thin-layer chromatography (TLC) and ultraviolet (UV) light spectrophotometric techniques also frequently used. The GC-MS analytical technique is mostly favoured due to the sensitivity and selectivity of the technique and the detection of individual compounds compared to the detection of a group of compounds associated with the other techniques [14]. However, GC-MS is limited to detection of volatile phytoconstituents, and such future work could deal with detection of components separated through their polarities using techniques such as liquid chromatography-mass spectra (LC-MS). It is further important to look at the relationship of the individual detected compounds from different locations using heat map and hierarchical clustering dendrograms.

**Figure 2:**
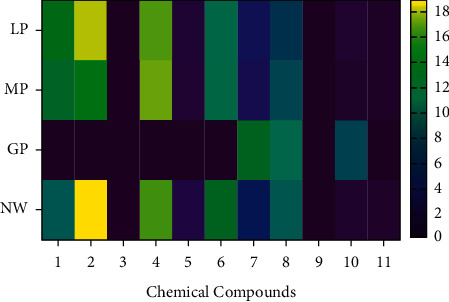
Heat map analysis of the relative abundance (% peak area) of phytoconstituents of *Aloe greatheadii* from four South African provinces. Chemical compounds: 1- pentadecane, 2- hexadecane, 3- 2-(((2-ethylhexyl)oxy)carbonyl)benzoic acid, 4- heptadecane, 5- ethyl 13-methyltetradecanoate, 6- octadecane, 7- eicosane, 8- heneicosane, 9- beta-sitosterol, 10- L-(+)-ascorbic acid 2,6-dihexadecanoate, and 11- tritetracontane. Abbreviations: LP- Limpopo Province, MP- Mpumalanga Province, GP- Gauteng Province, and NW- North-West Province.

The cluster heat map is a rectangular tiling of a data matrix with cluster trees appended to its margins. This can be within a relatively compact display area and facilitate comparison between rows (compounds), columns (location), and joint cluster structures. Heat map cluster analysis is most popular for its comparative nature of bringing large amounts of information into a visual small space to bring out coherent patterns in the data [15]. The % peak areas of the compounds detected in n-hexane extracts of samples from more than one province were calculated, and the results are expressed using a heat map analysis (Figure 2). The Limpopo, Mpumalanga, and North-West samples showed the presence of more compounds when compared to the sample from Gauteng. Hexadecane was the most abundant compound with peak areas of 18.9, 17.8, and 15.4% in North-West, Limpopo, and Mpumalanga, respectively. Hexadecane is a straight chain alkane with 16C (carbon atom) and is mainly a component of volatile oils. Heptadecane was the second most abundant compound with peak areas of 17.1, 16.8, and 16.6% for samples from Mpumalanga, Limpopo, and North-West, respectively. Heptadecane is a straight chain alkane with 17 C and is mainly a component of volatile oils [16]. The absence of hexadecane and heptadecane in the samples from Gauteng indicates how abiotic factors such as altitude, rainfall pattern, soil pH, and soil type affect the biochemical content of plants. Interestingly, eicosane and heneicosane (20 C and 21 C straight-chain alkanes) were detected in samples from Limpopo, Mpumalanga, Gauteng, and North-West. L-(+)-ascorbic acid 2, 6-dihexadecanoate (vitamin C) was detected with peak areas of 8.02, 1.07, 0.82, and 0.64% for samples from Gauteng, Limpopo, North-West, and Mpumalanga, respectively.

**Figure 3:**
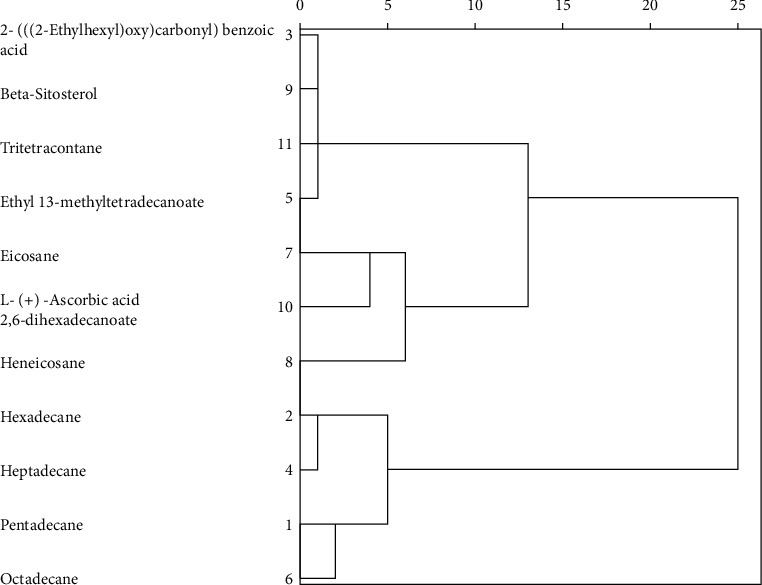
Hierarchical clustering dendrogram with clustering distance of phytochemical compounds of *Aloe greatheadii* from four South African provinces. The *Y*-axis represents the compounds detected, and the *X*-axis represents the clustering distance.

The apparent relationship of the different biochemical compounds from the four provinces was expressed using a hierarchical clustering dendrogram (Figure 3). Hierarchical clustering builds a cluster tree called a dendrogram to represent data with clustering distance, where each grouping links to two or more successor groups, with each group in the cluster tree containing a group of similar data [17]. The dendrogram results were able to group a number of compounds from leaves of *A. greatheadii* from four South African provinces into a number of clusters according to their concentration. The results were able to identify that the monoester of benzene (2-(((2-ethylhexyl) oxy) carbonyl) benzoic acid), phytosterol (beta-sitosterol), alkane (tritetracontane), and fatty acid methyl ester (ethyl 13-methyltetradecanoate) were closely related, thereby forming a cluster as characterized by the low clustering distance amongst all samples. The straight-chain alkanes pentadecane, hexadecane, heptadecane, and octadecane expressed a close relationship and formed a clustering group with a clustering distance of 5. Although eicosane, heneicosane, and L-(+)-ascorbic acid 2, 6-dihexadecanoate were detected in samples from all provinces, their concentrations varied substantially as expressed by the highest clustering distance amongst all samples. It was interesting to note that, within this cluster, eicosane and heneicosane levels were closely related, with them being straight-chain alkanes. The cluster with beta-sitosterol was closely related to the cluster with L-(+)-ascorbic acid 2,6-dihexadecanoate, forming a new cluster with a clustering distance of around 14. This newly formed cluster was significantly different from the straight-chain alkane cluster as it is shown by the clustering distance that is 5 times greater than the initial clustering group.

## 4. Conclusions

The comparative study on the leaves of *A. greatheadii* from four different provinces has been able to confirm that abiotic stress factors influence phytochemicals biochemical pathway composition. The heat map and hierarchical clustering dendrogram were further able to demonstrate how the number and levels of the chemical compounds have been affected. Therefore, abiotic stress factors have shown to influence phytochemical biochemical pathways and quantity. *A. greatheadii* plants can be selected based on their location, seemingly due to the variations that persists in their phytochemical content.

## Figures and Tables

**Table 1:** Abiotic stress factors (rainfall patterns, altitude, soil type, and soil pH) of the plant collection sites of the four provinces.

Location	Rainfall (annual average, mm)	Altitude (m)	Soil type	Soil pH
Limpopo	389	1329 m	Loam	5.17
Mpumalanga	490	1020 m	Loam	6.32
Gauteng	519	1246 m	Loam	5.98
North-West	540	1083 m	Clay	6.80

**Table 2:** GC-MS analysis of the presence and absence of compounds detected with 90% mass spectra similarity hit on the NIST08 library.

Compound name	Molecular weight	Molecular formula					Compound structure
			LP	MP	GP	NW	
Dodecanoic acid	200	C_12_H_24_O_2_	-	-	+	-	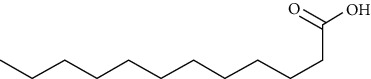
Pentadecane	212	C_15_H_32_	+	+	-	+	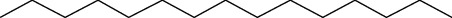
Hexadecane	226	C_16_H_34_	+	+	-	+	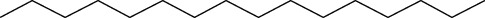
Tetradecanoic acid	228	C_14_H_28_O_2_	-	-	+	-	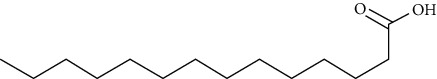
Heptadecane	240	C_17_H_36_	+	+	-	+	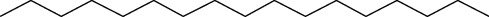
Octadecane	254	C_18_H_38_	+	+	-	+	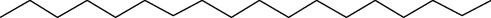
2-Pentadecanone, 6, 10, 14-trimethyl	268	C_18_H_36_O	+	-	+	+	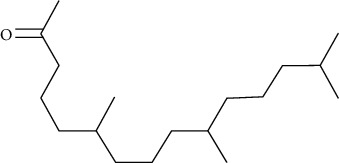
Oxacycloheptadecane-2, 9-dione	268	C_16_H_28_O_3_	-	+	-	-	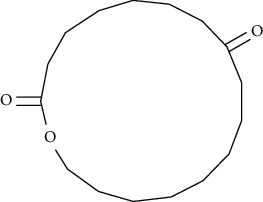
Methyl hexadecanoate	270	C_17_H_34_O_2_	-	-	+	-	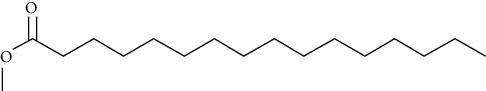
Ethyl 13-methyltetradecanoate	270	C_17_H_34_O_2_	+	+	-	+	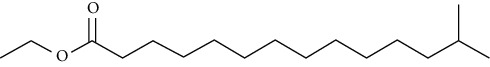
2-(((2-Ethylhexyl)oxy)carbonyl) benzoic acid	278	C_16_H_22_O_4_	-	+	-	+	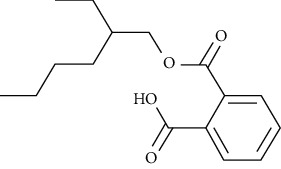
Eicosane	282	C_20_H_42_	+	+	+	+	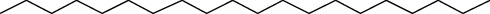
Ethyl hexadecanoate	284	C_18_H_68_O_8_	-	-	+	-	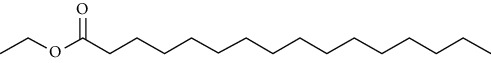
Dodecyl propan-2-yl sulfite	292	C_15_H_32_O_3_S	+	-	-	-	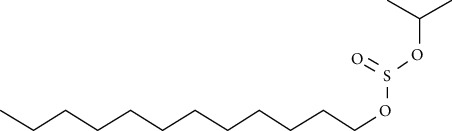
Heneicosane	296	C_21_H_44_	+	+	+	+	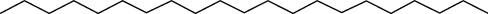
(Z)-icos-13-enoic acid	310	C_20_H_38_O_2_	+	-	-	-	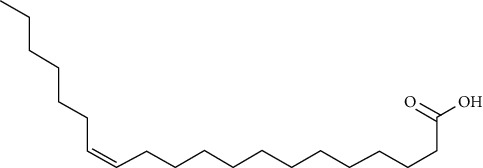
Eicosanoic acid	312	C_20_H_40_O_2_	-	+	-	-	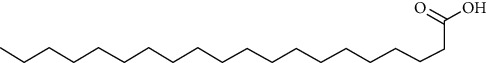
Beta-sitosterol	414	C_29_H_50_O	-	+	-	+	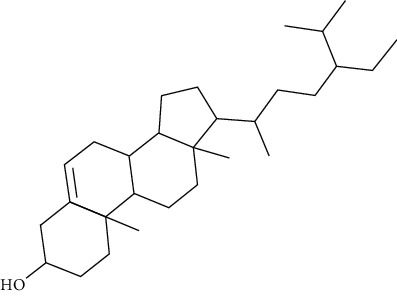
Tritetracontane	604	C_43_H_88_	-	+		+	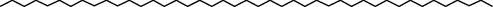
Tetratetracontane	618	C_44_H_90_	+	+	-	+	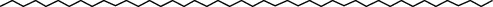
L-(+)-ascorbic acid 2, 6-dihexadecanoate	652	C_38_C_68_O_8_	+	+	+	+	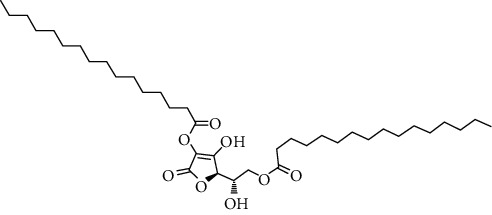
1, 54-Dibromotetrapentacontane	914	C_54_H_108_Br_2_	-	-	-	+	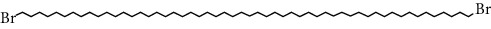

(+), presence; (-), absence.

## Data Availability

The supplementary data used to support the findings of this study are available from the corresponding author upon request.
